# Novel H_2_S donor proglumide-ADT-OH protects HUVECs from ox-LDL-induced injury through NF-κB and JAK/SATA pathway

**DOI:** 10.1515/med-2021-0287

**Published:** 2021-09-08

**Authors:** Xuelan Ou, Tianqin Xia, Chunyan Yang, Chunlei Yu, Shipeng Zhang, Rong Huang, Chuan Chen, Chunyang Zhou

**Affiliations:** Institute of Materia Medica, School of Pharmacy, North Sichuan Medical College, Nanchong 637100, Sichuan, China

**Keywords:** hydrogen sulfide donor, atherosclerosis, NF-кB, JAK/STAT, proglumide-(5-(4-hydroxyphenyl)-3*H*-1,2-dithiole-3-thione)

## Abstract

As a gaseous mediator, hydrogen sulfide (H_2_S) has many physiological effects and pathological effects in atherosclerosis. In recent years, many exogenous H_2_S donors have been synthesized to study atherosclerosis diseases. In this study, proglumide-(5-(4-hydroxyphenyl)-3*H*-1,2-dithiole-3-thione) (P-A) was synthesized as a H_2_S donor. The protective effect and mechanism of P-A on HUVEC that was injured by ox-LDL were detected. The HUEVCs were affected by 100 μmol/L P-A for 24 h; the release of H_2_S was the largest. After 100 μmol/L P-A acted on HUVEC damage model for 12 h, the cell proliferation activity was the best. The results showed that P-A can downregulate the expression of p-NF-кBp65 protein and reduce the amount of TNF-α and IL-6 and promote the formation of IL-10 by inhibiting the NF-кB pathway, and also induce the expression of superoxide dismutase (SOD) to protect HUVEC from ox-LDL injury. P-A can also regulate JAK/STAT pathway to reduce the expression of p-JAK2 protein and reduce the production of IL-6 and TNF-α. P-A has protective effect on HUVEC injured by ox-LDL, and the protective mechanism is related to the regulation of JAK/STAT pathway and NF-кB pathway.

## 1 Introduction

Atherosclerosis is a common cardiovascular disease caused by the interaction of environmental factors and genetic factors. The main manifestations of atherosclerosis include the lipid deposition of the intima, the infiltration of monocytes and macrophages, the formation of foam cells and fat veins, and the formation of fibrous plates caused by the migration and proliferation of vascular smooth muscle cells (VSMCs), which causes the hardening of the vascular wall and the stenosis of the functional cavity and the formation of thrombus [1].

H_2_S is a novel gas transmitter and has important physiological functions in atherosclerotic lesions [2]. The deficiency of H_2_S *in vivo* may be related to the early development of atherosclerotic lesions. On the contrary, an appropriate amount of hydrogen sulfide is helpful to delay atherosclerosis [3]. Thus far, more and more exogenous H_2_S donors have been created, including 5-(4-hydroxyphenyl)-3*H*-1,2-dithiole-3-thione (ADT-OH) [4]. ADT-OH is one of the most widely studied slow-releasing H_2_S donors.

The NF-кB plays an important role in inflammatory response, immune response, and cell growth and development [5]. H_2_S could decrease the production of TNF-α and IL-1β as well as leukocyte adhesion to the endothelium by inhibiting the activation of NF-кB [6]. Meanwhile, in the early atherosclerosis development process, TNF-α, IL-1β, IL-6, and IL-10 are closely related to the activation of JAK/STAT signaling pathway which is a signal transduction pathway that can be stimulated by cytokine and participates in the signal transduction and regulation process of various inflammatory and anti-inflammatory factors [7].

Proglumide could reduce the release of cytokines and inflammatory mediators by inhibiting the activation of NF-кB pathway in acute pancreatitis. Considering that one of the pathogeneses of atherosclerosis disease is related to the inflammation and the anti-inflammatory effect of proglumide, we combined proglumide with ADT-OH to create proglumide-(5-(4-hydroxyphenyl)-3*H*-1,2-dithiole-3-thione) (P-A). In this study, we proved that P-A is a novel slow-releasing H_2_S donor and shows anti-atherosclerotic effect on the HUVECs injured model by inhibiting the activation of JAK/STAT pathway and NF-кB pathway.

## 2 Materials and methods

### 2.1 Synthesis of P-A

#### 2.1.1 Materials

5-(4-Methoxyphenyl)-1,2-dithiole-3-thione (ADT); proglumide; anhydrous pyridine hydrochloride; dichloromethane (DCM); *N*,*N*-dimethyl-4-pyridinamine (DMAP); sodium hydroxide (NaOH); *N,N*′-dicyclohexylcarbodiimide (DCC). Chemicals were purchased from Sigma-Aldrich in St. Louis, USA.

#### 2.1.2 Step 1

**Figure A1:**
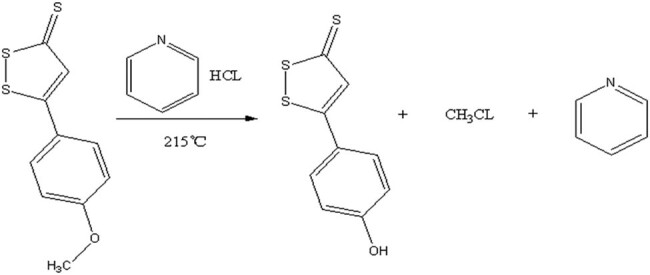
Reaction equation for the synthesis of ADT-OH compounds.

Synthesis of ADT-OH (Figure A1, Table A1). Dry ADT (10.59 g) was mixed with anhydrous pyridine hydrochloride (330.16 g) and heated to 215°C in oil bath (LABCONCO, Chengdu, China). The melting of solid marks the beginning of the reaction, and the reaction is terminated after 60 min. After the product was cooled to 25°C, 10 mL 1 mol/L HCl solution (Kelong, Chengdu, China) was added to dissolve the product and then the solution was filtered. After the filtrate is removed, the product is washed to neutral by distilled water. And then after vacuum filtration, the residue was retained. The ADT-OH was obtained by recrystallization of anhydrous ethanol (Sigma-Aldrich, St. Louis, USA). The productivity of ADT-OH was 86.32%, with a total of 9.1382 g.

#### 2.1.3 Step 2

**Figure 1:**
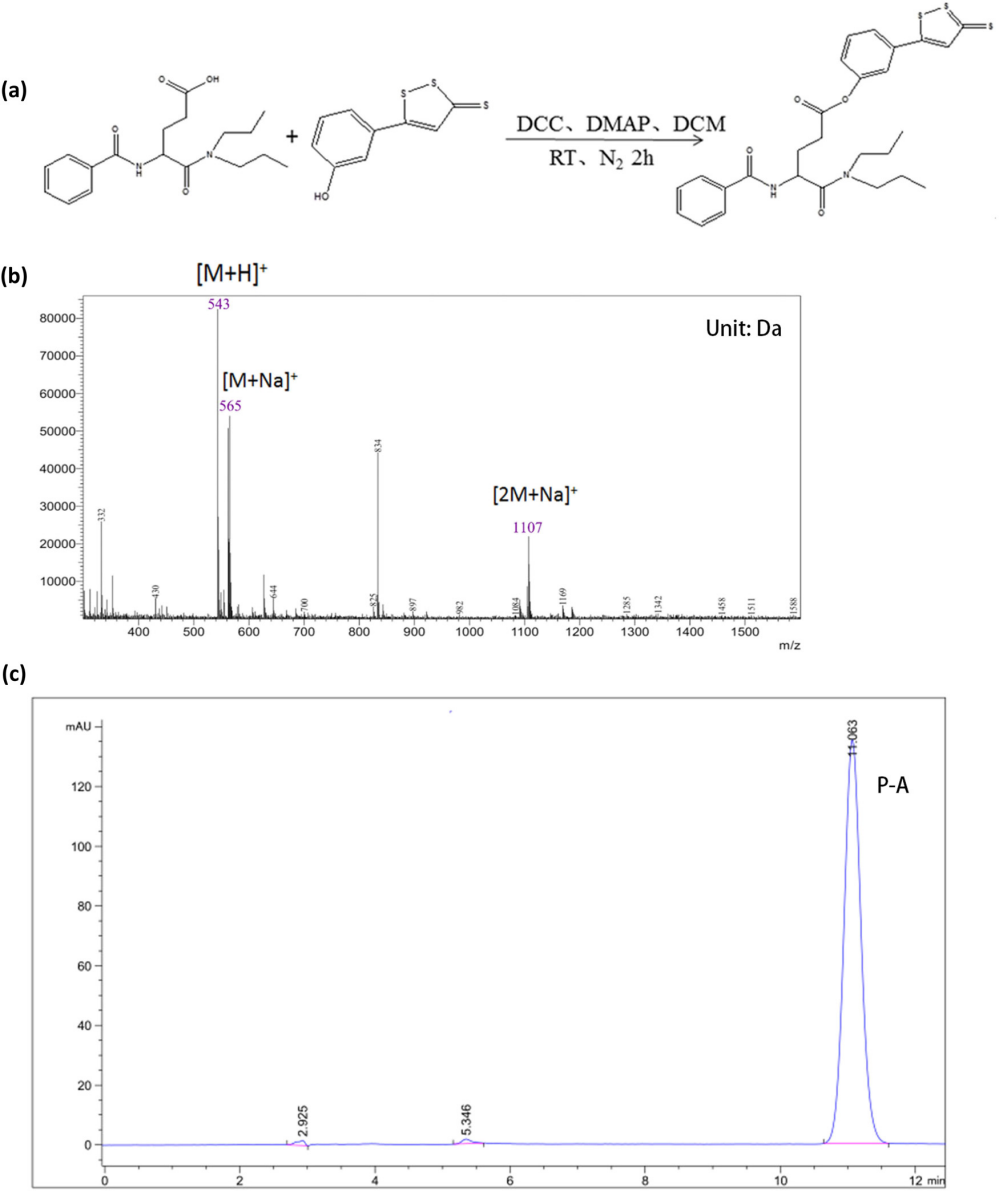
P-A was successfully synthesized. (a) The method of synthesizing P-A. (b) MS of P-A. The molecular weight of P-A was 542 by MS. The molecular weight was 543, indicating the combination of P-A and H. The molecular weight was 565, indicating the combination of P-A and Na^+^. The molecular weight was 1,107, indicating the combination of 2 molecules of P-A and Na^+^. (c) P-A has higher purity and less impurity content, as well as a single peak was showed by HPLC.

Synthesis of P-A (Figure 1a, Table A2). After the reaction was stopped, a small amount of 1 mol/L NaOH solution (∼120 µL) was added to the reaction termination system until the color of the pH test paper displayed 7. After vacuum filtration for 2–3 times, filter the residue and filtrate. The filtrate is added to water in the separation funnel and layered, the water layer was removed, and the dichloromethane layer was retained. The residual water in the dichloromethane layer was removed by adding anhydrous sodium sulfate (Kelong, Chengdu, China). After vacuum filtration 2–3 times, the residue was filtered and the filtrate was left. The products were obtained after drying with rotary evaporator (LABCONCO, Chengdu, China) (LABCONCO, Chengdu, China) at 50°C. The product is dissolved in anhydrous ethanol. The product was recrystallized in the refrigerator at −20°C for one night. After vacuum filtration for 2–3 times, the product was retained. The oil pump (LABCONCO, Chengdu, China) is evacuated to remove the product water and organic solvent to obtain P-A.

### 2.2 Detection of H_2_S releasing

HUVECs cells (ATCC, Manassas, VA, USA) were incubated in 12-well plates, and four microporous filtering films of 0.22 µm were adhered to the inner side of the 12-well plates of each hole to set up the filter membrane adsorption device. After 500 µL 1% (g/100 mL) zinc acetate (Kelong, Chengdu, China) solution was added to each filter membrane, the P-A solutions were added in HUVECs. The filter membranes were collected after P-A acted on HUVEC for 1, 3, 6, 12, 24, and 48 h, respectively. Then the filter membrane was soaked in 2.5 mL ultrapure water. The release of H_2_S was detected by methylene blue spectrophotometry (LABCONCO, Chengdu, China) at 670 nm, and the Na_2_S standard curve was drawn according to the OD.

### 2.3 Establishment of ox-LDL-induced HUVECs injured model

HUVECs were damaged by 80 µg/mL ox-LDL (Sigma-Aldrich, St. Louis, USA) for 24 h. The oil red O staining method was used to judge whether the HUVEC had been damaged.

### 2.4 CCK-8 assay

HUVECs were seeded in 96-well plates and cultured for overnight at 37°C. HUVECs were induced by 80 µg/mL ox-LDL for 24 h, then the P-A (12.5, 25, 50, 100, and 200 µmol/L) was acted on HUVECs for 24 h. Then cell proliferation was detected by CCK8 kits (Boster Biotechnology, Chengdu, China) according to the manufacturer’s protocol. Absorbance was determined at the 450 nm by enzyme-linked immunosorbent assay reader (LABCONCO, Chengdu, China).

### 2.5 ELISA assay of IL-6, IL-10, and TNF-α

After the HUVECs were injured for 24 h by 80 µg/mL ox-LDL in 12-well plates, the HUVECs were treated with 100 μmol/L P-A for 12 h. The supernatant from each well was collected and used to detect the secretion of IL-6, IL-10, and TNF-α by ELISA assay with commercial ELISA kits of IL-6, IL-10, and TNF-α (Boster Biotechnology, Chengdu, China) according to the manufacturer’s protocol. In the AG490and PDTC pretreated assay, AG490 and PDTC were added in HUVECs, respectively, for 1 h before 80 µg/mL ox-LDL induced HUVEC for 24 h. All other methods were the same as described above.

### 2.6 Determination of intracellular SOD

After being injured by 80 µg/mL ox-LDL for 24 h, HUVECs were treated with 100 μmol/L P-A for 12 h. Then HUVECs were collected to lysis at 4°C in RIPA buffer (Yiyuan Biotechnology, Guangzhou, China). The lysate was clarified by centrifugation at 12,000 rpm for 15 min at 4°C. Protein concentration of HUVEC lysate was determined by BCA assay kits (Yiyuan Biotechnology, Guangzhou, China). The activity of intracellular SOD was determined by SOD assay kits (Yiyuan Biotechnology, Guangzhou, China) according to the manufacturer’s protocol.

### 2.7 Protein expression by western blotting

p-NF-кBp65, p-JAK2, p-STAT3, NF-кBp65, JAK2, STAT3 antibody, and goat anti-rabbit IgG were from Cell Signaling Technology, China. The total protein was extracted from HUVECs according to the standard procedures. Protein samples (40 μg) were separated by 10% SDS-PAGE (sodium dodecyl sulfate polyacrylamide gel electrophoresis) and then transferred into PVDF membranes (Yiyuan Biotechnology, Guangzhou, China). The membrane was blocked with 5% nonfat dry milk solutions. After washing the PVDF membranes with TBST (tris buffered saline tween), the PVDF membranes were incubated overnight at 4°C with the above antibody, respectively. It was followed by secondary antibody for 2 h with goat anti-rabbit IgG. After washing, the membrane was developed with ECL kit (Yiyuan Biotechnology, Guangzhou, China) and detected with VILBER Fusion FX5 system (VILBER, Guangzhou, China).

### 2.8 Statistical analysis

All data were analyzed with GraphPad Prism 5 (GraphPad, San Diego, USA) and were presented as the mean ± SD. For all tests, *P* < 0.05 was considered statistically significant.

**Ethical approval:** The conducted research is not related to either human or animal use.

## 3 Results

### 3.1 P-A was synthesized successfully

As proved by ^1^H-NMR (Table 1), MS (Figure 1b), and HPLC (Figure 1c), P-A was successfully synthesized and used for subsequent experiments.

**Table 1:** ^1^H-NMR spectrum (400 MHz, DMSO-d_6_) of P-A

Compound	NMR (dppm)
C_27_H_34_N_2_O_4_S_3_	*δ*_H_ 7.98(d, *J* = 8.8 Hz, H-3‴, 5‴), 7.92(d, *J* = 7.6 Hz, H-2′, 6′), 7.82(s, H-4‴′), 7.54(t, *J* = 6.8, 7.8 Hz, H-4′), 7.47(t, *J* = 7.6, 7.8 Hz, H = 3′, 5′), 7.32(d, *J* = 8.8 Hz, H-2‴, 6‴), 3.07(m, H-4), 2.73(m, H-1″), 2.05(m, H-2), 1.61(m, H-3), 1.47(m, H-2″), 0.83 (t, *J* = 7.6, 7.2 Hz, H-3″)

### 3.2 P-A is a slow-releasing H_2_S donor

**Figure 2:**
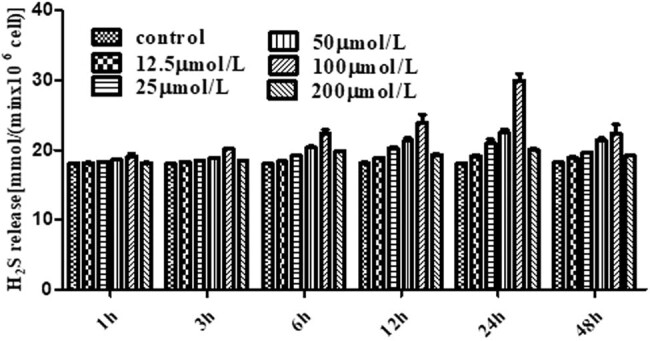
HUVECs produce H_2_S after P-A was added in cells. The release of H_2_S of different concentrations P-A acted on HUVECs at different time. The release of H_2_S was the largest after the HUEVCs were affected by 100 μmol/L P-A for 24 h. (Mean ± SD, *n* = 3).

The H_2_S productivity of P-A was analyzed in HUEVCs, and we found that the release of H_2_S (18–32 mmol/L) from P-A increased in a time- and concentration-dependent manner, generally. However, the release rate decreased after the incubation time reached 24 h or the concentration reached 100 μmol/L (Table 2, Figure 2).

**Table 2:** The release of H_2_S from P-A

Time (h)	Control	12.5 μmol/L	25 μmol/L	50 μmol/L	100 μmol/L	200 μmol/L
1	18.027 ± 0.038	18.160 ± 0.046	18.333 ± 0.013	18.577 ± 0.049	19.063 ± 0.347^Δ^	18.171 ± 0.031^Δ^
3	18.020 ± 0.005	18.243 ± 0.056	18.414 ± 0.012	18.860 ± 0.032	20.177 ± 0.036^Δ^	18.425 ± 0.007^&^
6	18.012 ± 0.015	18.387 ± 0.020	19.182 ± 0.037*	20.302 ± 0.227^#^	22.328 ± 0.597^Δ^	19.807 ± 0.111^&&^
12	18.126 ± 0.067	18.852 ± 0.076	20.202 ± 0.203*	21.286 ± 0.433^#^	23.938 ± 1.130^Δ^	19.313 ± 0.070^&^
24	18.075 ± 0.082	19.071 ± 0.189^∼^	20.893 ± 0.596*	22.443 ± 0.509^#^	29.927 ± 0.997^ΔΔ^	20.102 ± 0.058^&&^
48	18.234 ± 0.086	18.881 ± 0.074	19.664 ± 0.021	21.391 ± 0.266^#^	22.339 ± 1.323^Δ^	19.132 ± 0.021^&^

^*^P < 0.05 vs 12.5 μmol/L; ^#^ *P* < 0.05 vs 25 μmol/L; ^Δ^ *P* < 0.05 ^ΔΔ^ *P* < 0.01 vs 50 μmol/L; ^&^ *P* < 0.05 ^&&^ *P* < 0.01 vs 100 μmol/L; ^&^ *P* < 0.05 ^&&^ *P* < 0.01 vs 200 μmol/L; ^∼^ *P* < 0.05 vs control.

### 3.3 P-A reliefs ox-LDL-induced HUVECs injury

**Figure 3:**
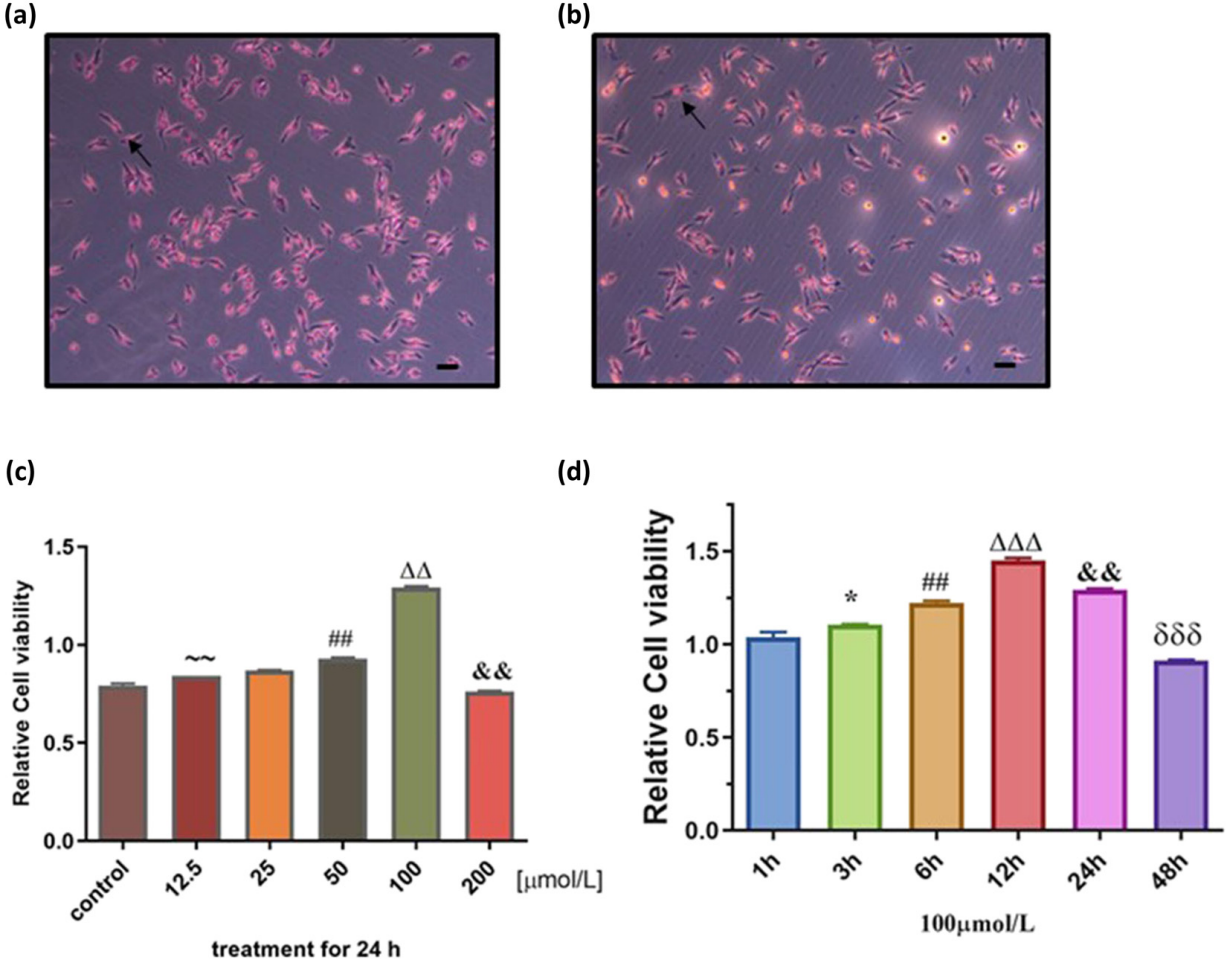
The cell proliferation of different concentrations of P-A acted on HUVEC at different time. (a) Representative images of HUVECs. (b) Representative images of ox-LDL-induced HUVECs. After the HUVECs were induced by 80 μg/mL ox-LDL for 24 h, the cells had been damaged. (c) The cell proliferation activity increased with time in a concentration-dependent manner in 0–24 h. ^∼∼^ *P* < 0.01 vs control; ^##^ *P* < 0.05 vs 25 μmol/L; ^ΔΔ^ *P* < 0.01 vs 50 μmol/L; ^&&^ *P* < 0.01 vs 100 μmol/L. (d) After 100 μmol/L P-A acted on HUVEC 12 h, the cell proliferation activity was the largest. ^*^ *P* < 0.05 vs 1 h; ^##^ *P* < 0.05 vs 3 h; ^ΔΔΔ^ *P* < 0.001 vs 6 h; ^&&^ *P* < 0.01 vs 12 h; ^δδδ^ *P* < 0.001 vs 24 h. (Mean ± SD, *n* = 3).

To test the protective effect of P-A on vein endothelial cells, we established the ox-LDL-injured HUVECs *in vitro* model. After the HUVECs were induced by 80 μg/mL ox-LDL for 24 h, oil red O staining showed that a large number of red dye particles appeared in the cells (Figure 3a and b). This phenomenon indicated that HUVECs had formed damage which causes the oil red O enter into the cell and dissolve in the lipid. Then we treated the ox-LDL-injured HUVECs with P-A, as shown in Figure 3; the cell proliferation activity increased with time in a concentration-dependent manner in 24 h, but only up to 100 μmol/L P-A concentration (Figure 3c). When the concentration of P-A reached to 200 µmol/L, the declined cell viability indicated that P-A produces cytotoxicity at very high concentration above 100 µmol/L. (Figure 3c). After 100 μmol/L P-A acted on HUVECs damage model for 12 h, the cell proliferation activity was the best (Figure 3d).

### 3.4 P-A regulates the expression of IL-6, IL-10, TNF-α, and SOD

Compared with normal cell control group, the amount of IL-6, TNF-α, and IL-10 in the HUVEC injury model group increased significantly (*P* < 0.01). As shown in Table 3, compared with the HUVEC damage model group, the secretion of IL-6 and TNF-α reduced significantly after treated with P-A as well as positive control NaHS and ADT-OH (*P* < 0.01), while the secretion of IL-10 increased significantly (*P* < 0.01).

**Table 3:** Effects of P-A, NaHS, Proglumide, and ADT-OH on IL-6, IL-10, and TNF-α in HUVEC damage model (\bar{x}]± S, pg/mL, *n* = 5)

Group	IL-6	IL-10	TNF-α
Cell control	10.25 ± 0.5848	4.39 ± 0.0058	10.25 ± 0.5839
Model	75.03 ± 2.442**	9.486 ± 0.5332**	35.28 ± 2.040**
NaHS	29.64 ± 1.839^#⊿⊿^	17.66 ± 1.471^⊿⊿^	19.99 ± 0.8625^⊿⊿^
P-A	18.55 ± 0.4347^⊿⊿^	22.25 ± 1.786^⊿⊿^	18.61 ± 1.258^⊿⊿^
Proglumide	50.56 ± 2.4130^#⊿⊿^	13.50 ± 1.056^#⊿⊿^	31.99 ± 0.9677
ADT-OH	26.07 ± 1.718^⊿⊿^	9.384 ± 0.3275	23.19 ± 1.628^⊿⊿^

^**^P < 0.01 vs cell control; ^#^ *P* < 0.05 vs P-A; ^⊿⊿^ *P* < 0.01 vs model.

**Figure 4:**
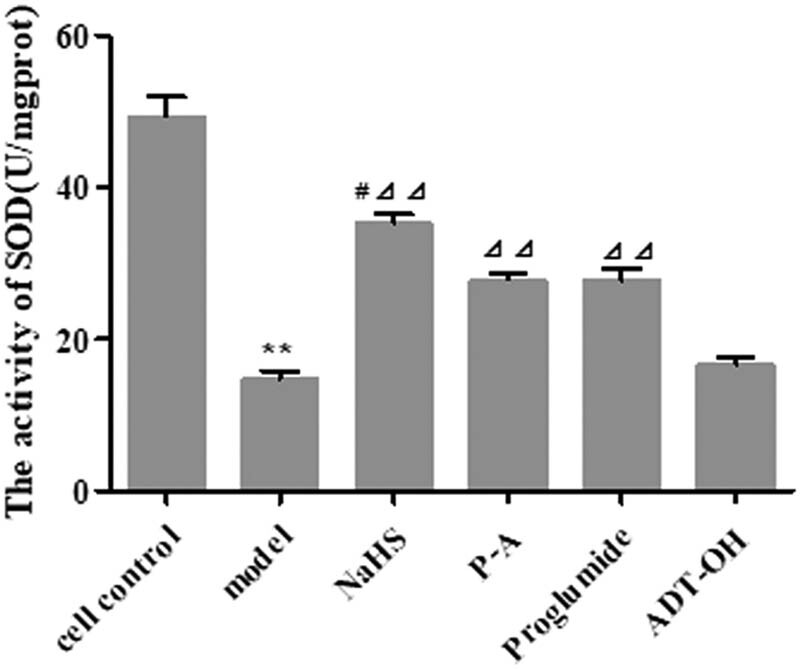
Effect of P-A acted on the activity of SOD in HUVEC damage model. (Mean ± SD, *n* = 4) **P < 0.01 vs cell control; ^⊿⊿^ *P* < 0.01 vs model; ^#^ *P* < 0.05 vs P-A.

SOD can regulate the level of superoxide anion in the vascular wall and alleviate the oxidative damage of oxygen-free radicals to endothelial cells, as well as protect endothelial cells from atherosclerosis [8]; therefore, we analyzed the SOD level after P-A treatment. As shown in Figure 4, compared with normal cell control group, the activity of SOD in the HUVEC injury model group decreased significantly (*P* < 0.01). Compared with the HUVEC damage model group, the activity of SOD in the NaHS group, P-A group, and proglumide group increased significantly (*P* < 0.01).

### 3.5 P-A regulates expression of IL-6, IL-10, and TNF-α through NF-кB and JAK/SATA pathway

**Figure 5:**
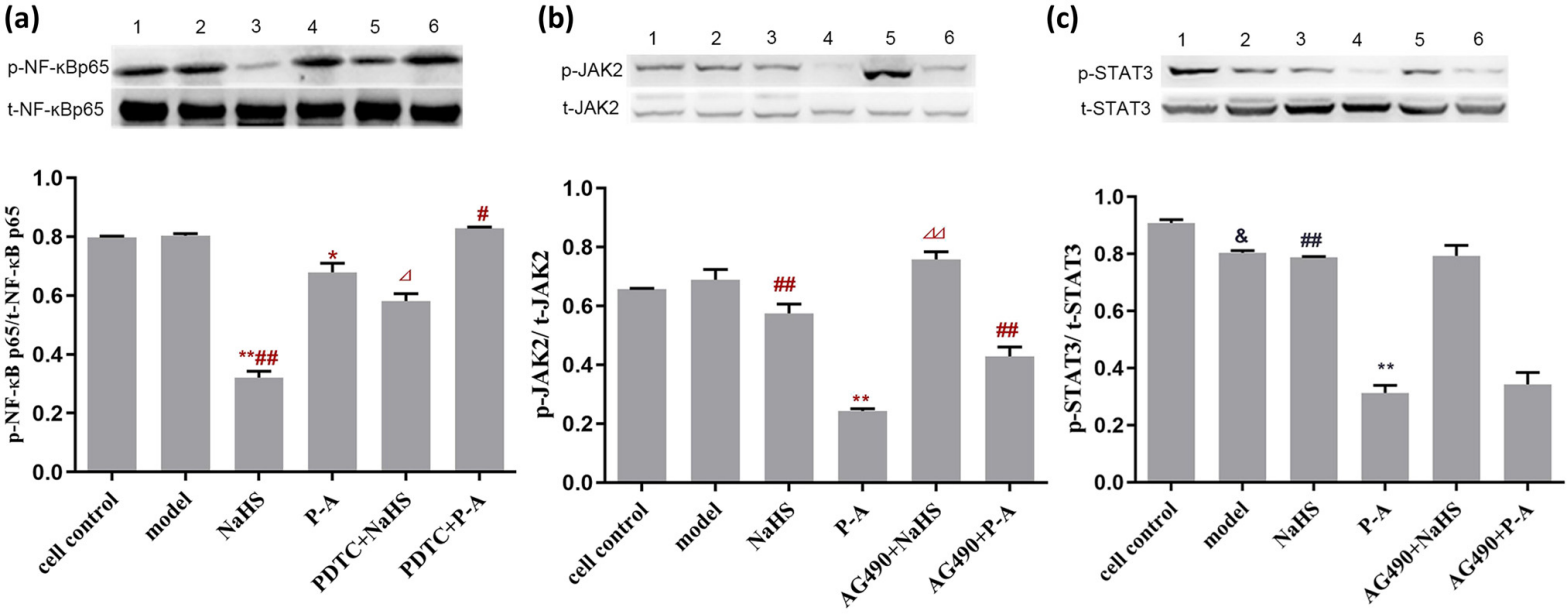
P-A regulates expression of IL-6, IL-10, and TNF-α through NF-кB and JAK/SATA pathways. (a) The expression of p-NF-кB p65 protein in HUVEC damage model. (1) cell control; (2) model; (3) NaHS; (4) P-A; (5) PDTC + NaHS; (6) PDTC + P-A. ^*^ *P* < 0.05 ^**^ *P* < 0.01 vs model; ^#^ *P* < 0.05 ^##^ *P* < 0.01 vs P-A; ^⊿^ *P* < 0.05 vs NaHS. Mean ± SD, *n* = 3. (b) The expression of p-JAK2 protein in the HUVEC damage model. (1) cell control; (2) model; (3) NaHS; (4) P-A; (5) AG490 + NaHS; 6: AG490 + P-A. (c) The expression of p-STAT3 protein in the HUVEC damage model. (1) cell control; (2) model; (3) NaHS; (4) P-A; (5) AG490 + NaHS; 6: AG490 + P-A. ^**^ *P* < 0.01 vs model; ^##^ *P* < 0.01 vs P-A; ^⊿⊿^ *P* < 0.01 vs NaHS. Mean ± SD, *n* = 3.

Compared with P-A group, the expression of p-NF-кBp65 protein increased in the PDTC + P-A group (Figure 5a, *P* < 0.05); the expression of p-NF-кBp65 protein reduced and the expression of p-JAK2 protein as well as the expression of p-STAT3 protein increased in NaHS group (Figure 5a–c); the expression of p-JAK2 protein increased in the AG490 + P-A group(Figure 5b, *P* < 0.01). Compared with NaHS group, the expression of p-NF-кBp65 protein increased in the PDTC + NaHS group (Figure 5a, *P* < 0.05); the expression of p-JAK2 protein increased in the AG490 + P-A group (Figure 5b, *P* < 0.01).

As shown in Table 4, compared with NaHS group, the amount of IL-6 and TNF-α increased significantly as well as the amount of IL-10 reduced significantly in the PDTC + NaHS group in the PDTC + NaHS group (*P* < 0.05). Compared with P-A group, the amount of IL-6 increased significantly and the amount of IL-10 reduced significantly in the PDTC + P-A group and AG490 + P-A group (*P* < 0.05); the amount of TNF-α increased significantly in the PDTC + P-A group (*P* < 0.05). Compared with Proglumide group, the amount of IL-10 reduced significantly in the PDTC + Proglumide group (*P* < 0.05). Compared with ADT-OH group, the amount of IL-6 increased significantly (*P* < 0.05).

**Table 4:** Detection of IL-6, IL-10, and TNF-α after adding AG490 and PDTC in HUVEC damage model (\bar{x}] ± S, pg/mL, *n* = 5)

Group	IL-6	IL-10	TNF-α
NaHS	29.64 ± 1.839	17.66 ± 1.471	19.99 ± 0.8625
PDTC + NaHS	40.74 ± 2.177*	13.03 ± 1.525*	27.02 ± 0.8608*
AG490 + NaHS	37.88 ± 1.289	14.77 ± 0.8331	24.21 ± 0.9830
P-A	18.55 ± 0.4347	22.25 ± 1.786	18.61 ± 1.258
PDTC + P-A	30.48 ± 2.088^#^	14.73 ± 1.543^#^	28.00 ± 1.488^#^
AG490 + P-A	35.51 ± 1.959^#^	16.85 ± 1.355^#^	23.65 ± 0.6302
Proglumide	50.56 ± 2.4130	13.50 ± 1.056	31.99 ± 0.9677
PDTC + Proglumide	51.34 ± 1.837	9.562 ± 0.4209^⊿^	30.38 ± 1.408
AG490 + Proglumide	58.97 ± 2.678	12.96 ± 1.272	30.43 ± 1.479
ADT-OH	26.07 ± 1.718	9.384 ± 0.3275	23.19 ± 1.628
PDTC + ADT-OH	32.12 ± 1.815^	9.537 ± 0.4209	27.01 ± 2.950
AG490 + ADT-OH	29.87 ± 0.4123	9.318 ± 0.4197	27.31 ± 1.596

*P < 0.05 vs NaHS; ^#^ *P* < 0.05 vs P-A; ^⊿^ *P* < 0.05 vs Proglumide; ^*P* < 0.05 vs ADT-OH.

## 4 Discussion

The current research on H_2_S presents the trend of cross-disciplinary research in pharmacology, physiology, chemistry, biology, materials science, etc. [9]. In addition to the endogenous H_2_S and the traditional hydrogen sulfide donor NaHS, more and more exogenous hydrogen sulfide donor [10] and some sulfur compounds extracted from natural plants have also been widely studied [11]. In this study, a novel hydrogen sulfide donor P-A was successfully synthesized as proved by ^1^H-NMR and MS. The demethylation reaction of ADT is the key in the whole synthesis reaction. The purity of the P-A will be affected by the purity of the ADT-OH. The addition of DCC in this system can activate carboxyl. After the reaction was stopped, adding NaOH solutions to the reaction system to pH 7 can wash out some acidic by-products and can also adjust the reaction system pH to the neutral to avoid the degradation of the products. The chemical synthesis method in this experiment is simple and the reaction conditions are mild, while the post-processing is also simple. The product is easy to be purified through recrystallization. The study shows that the synthetic method in this experiment can be used to obtain the target product P-A, which also provides valuable reference for the synthesis of other H_2_S donors in the future.

With the increased amount of P-A used for treating HUVECs, the release of hydrogen sulfide and the cell proliferation gradually increased. However, excessive P-A has cytotoxic effect on cells; the proliferation and release of hydrogen sulfide were inhibited.

Vascular endothelial cells, smooth muscle cells, and macrophages can secrete interleukin at different stages of inflammation [12]. In our study, we found that intracellular triglyceride and cholesterol metabolism disorder cause lipid aggregation to damage endothelial cells after the HUVEC was injured by ox-LDL, and the inflammatory reaction started at the same time, as well as the secretion of inflammatory factors TNF-α, IL-6, and anti-inflammatory factor IL-10 increased. IL-6 can cause chronic inflammation and magnify acute inflammatory response to some extent and promote the release of some chemokines and reactive oxygen species to participate in and further aggravate the atherosclerosis process [13]. TNF-α is present in atherosclerotic plaques, which stimulates the production of inflammatory factors and directly promotes the development of inflammation. IL-10 has the functions of anti-inflammatory for the atherosclerosis disease [14].

The release of TNF-α, IL-6, and IL-10 can activate NF-кB to promote the production of inflammatory factors such as IL-8, which further aggravates the inflammatory reaction in the atherosclerosis process [15,16]. Our study shows that P-A and ADT-OH can significantly reduce the secretion of IL-6 and TNF-α in the HUVEC damage model. It is indicated that P-A reduces the secretion of IL-6 and TNF-α, which is related to the structure of ADT-OH. P-A and proglumide can significantly increase the secretion of IL-10 in the HUVEC damage model. It is indicated that P-A increases the secretion of IL-10, which is related to the structure of proglumide.

H_2_S can inhibit the expression of intercellular adhesion molecule-1 mediated by NF-кB pathway in HUEVC and induce the expression of SOD in endothelial cells at the same time [17]. We found that P-A can significantly increase the activity of SOD in the experimental ox-LDL-affected HUVECs, which is related to the structure of proglumide.

As the main transcription factor of the inflammatory response, NF-кB can be activated by IL-6, TNF-α, CRP, and so on, which participates in the whole process of atherosclerosis [18,19]. JAK/STAT signal transduction pathway is activated after the JAK2 phosphorylation. Inhibition of JAK2 activity can inhibit STAT3 phosphorylation, thereby inhibiting the production of IL-6, IL-8, TNF-α, and other inflammatory factors. Blocking the JAK/STAT signal pathway can effectively prevent the occurrence and aggravation of atherosclerosis diseases [20]. Our study found that P-A can downregulate the expression of p-NF-кBp65 protein and reduce the production of TNF-α and IL-6 and promote the formation of IL-10 by inhibiting the NF-кB pathway, and also induce the expression of SOD in HUVEC damage model to protect HUVEC from ox-LDL. P-A can also regulate JAK/STAT signal transduction pathway to reduce the expression of p-JAK2 protein and reduce the cell proliferation.

However, there is no direct evidence showing that P-A protects HUVEC from ox-LDL damage only through the NF-кB pathway and JAK/STAT signaling pathway. Other associated signaling pathways may also play important roles in protecting HUVECs from ox-LDL damage. Based on the existing basis, the further study of the P-A is needed to find the downstream targets and genes of NF-кB pathway and JAK/STAT signaling pathway protecting HUVECs, as well as the receptors of IL-6, IL-10, and TNF-α mediated by NF-кB signaling pathway and JAK/STAT signaling pathway, and other related signaling pathways and indicators.

In conclusion, P-A has the protective effect for experimental ox-LDL-affected HUVEC, and the protective mechanism is related to the regulation of JAK/STAT pathway and NF-кB pathway to some extent. What’s more, our study provides direct evidence that JAK/STAT pathway and NF-кB pathway participate in the atherosclerosis process.

## Appendix

**Table A1:** Synthesis of ADT-OH

Reagent	C_10_H_8_OS_3_ (ADT)	C_5_H_6_NCl
*M* (g/mol)	240.36	115.56
*m* (g)	10.5853	50.01
*n* (mol)	0.44	0.4327
Melting point (℃)	110	−41.6
Boiling point (℃)	—	115.2
Solvent	—	
Reaction temperature (℃)	215	
Reaction time(min)	60

**Table A2:** Synthesis of P-A

Reagent	*M* (g/mol)	*m* (g)	*n* (mmol)
Proglumide	334.42	1.338	4
ADT-OH	226.34	0.995	4.4
DCC	206.33	0.992	4.8
DAMP		0.066	
DCM		100 mL	
Reaction conditions		RT, 4h, N_2_	
